# On the Effects of Different *trans* and *cis* Populations in Azobenzene Liquid Crystal Elastomers: A Monte Carlo Investigation

**DOI:** 10.1021/acsapm.3c00361

**Published:** 2023-07-26

**Authors:** Gregor Skačej, Lara Querciagrossa, Claudio Zannoni

**Affiliations:** † Faculty of Mathematics and Physics, University of Ljubljana, SI-1000 Ljubljana, Slovenia; ‡ Dipartimento di Chimica Industriale “Toso Montanari”, Università di Bologna, Viale Risorgimento 4, I-40136 Bologna, Italy; § CINECA, Via Magnanelli 6/3, I-40033 Casalecchio di Reno, Italy

**Keywords:** liquid crystal elastomers, azobenzene, actuation, Gay−Berne potential, Monte Carlo simulations

## Abstract

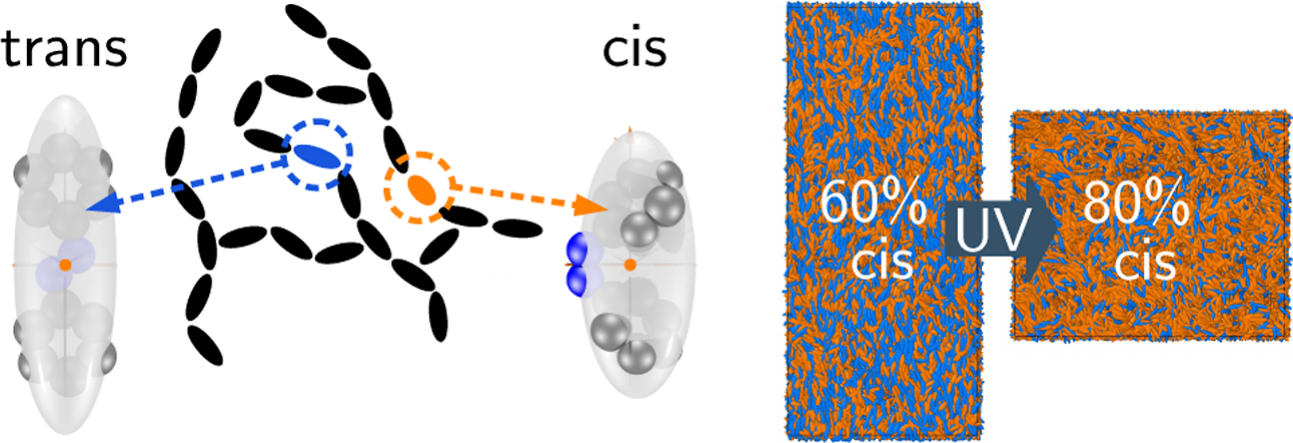

We investigate main-chain liquid crystal elastomers (LCEs) formed by photoresponsive azobenzene units with different populations of *trans* and *cis* conformers (from fully *trans* to fully *cis*). We study their macroscopic properties as well as their molecular organization using extensive Monte Carlo simulations of a simple coarse-grained model where the *trans* and *cis* conformers are represented by soft-core biaxial Gay–Berne particles with size and interaction energy parameters obtained by fitting a bare bone azobenzene moiety represented at atomistic level. We find that increasing the fraction of *cis* conformers, as could be obtained by near-UV irradiation, shifts the nematic–isotropic transition to a lower temperature, consistently with experiment, while generating internal stress in a clamped sample. An analysis of pair distributions shows that the immediate surroundings of a bent *cis* molecule are slightly less dense and more orientationally disordered in comparison with that of a *trans* conformer. Comparing nematic and smectic LCEs, actuation in the smectic phase proved less effective, disrupting the smectic layers to some extent but preserving orientational order of the azobenzene moieties.

## Introduction

Stimuli-responsive materials are an important class of smart materials capable of responding to an externally applied trigger through an observable property change. These triggers can include temperature variation, application of an external electric or magnetic field, and irradiation with light, of interest here.^1^ The latter applies, for example, to photoresponsive polymers^2,3^ containing azobenzene moieties whose molecular shape anisotropy changes significantly upon irradiation with near-ultraviolet (UV) light causing a *trans* to *cis* isomerization.^4,5^ If these moieties are themselves mesogenic and exhibit nematic or smectic phases,^6,7^ it is found that their orientational, liquid-crystalline ordering can be decreased or destroyed upon irradiation with light even at constant temperature (with an effect similar to that of increasing temperature).^8−14^ Somewhat amazingly the conformational changes take place even in very viscous glassy materials, possibly through photofluidization,^15^ although the issue is still debated.^16^ Photoresponsive liquid crystal elastomers (LCEs), characterized by a pronounced strain-alignment coupling, can be obtained when azobenzenes are covalently linked to a cross-linked polymeric network^1,17−19^ or even just dissolved as guest in the LCE matrix.^20^ Such materials can exhibit significant elastic deformations upon phase transitions between the orientationally ordered liquid crystalline state and the disordered isotropic phase and can thus act as photoactuators converting light energy into mechanical work.^20−23^ In view of this, photoresponsive LCEs are promising candidates for a variety of applications,^24^ including sensors and actuators,^1,25−27^ photomechanical devices,^19^ morphing surfaces,^22^ soft robotics,^28,29^ and more, also thanks to advances in chemical synthesis.^30^

**Figure 1:**
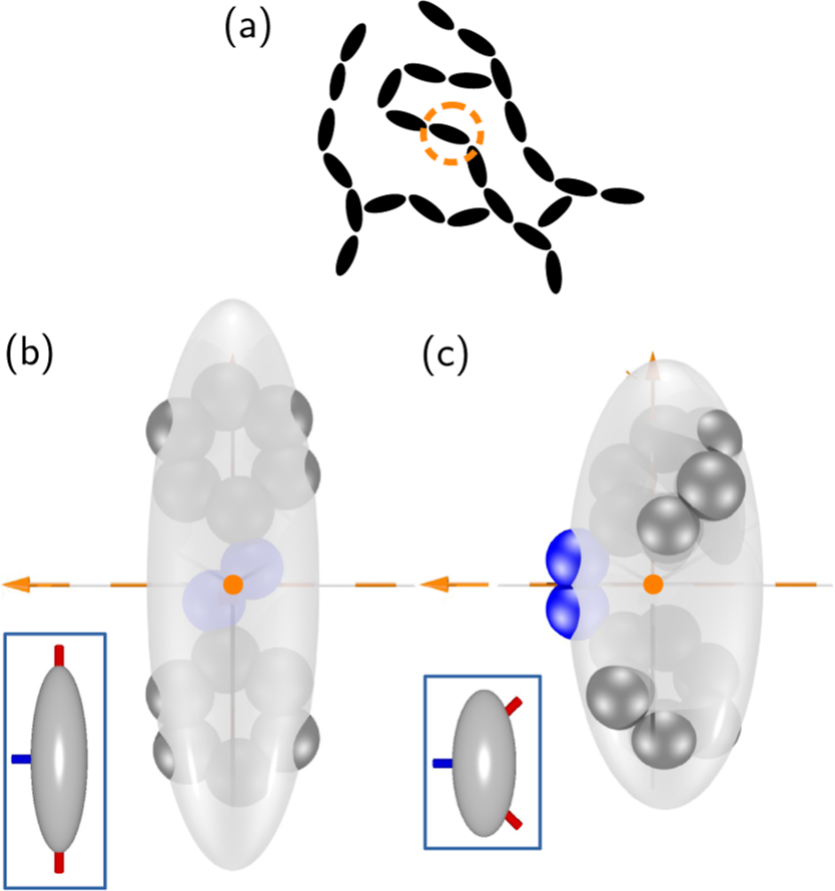
(a) Sketch of the typical main-chain LCE network topology. The dashed circle indicates one of the *trans* or *cis* azobenzene units whose united-atom representation is shown together with their coarse-grained GB ellipsoids in (b), *trans*, and (c), *cis*. The grey and blue spheres represent the −CH– groups and the nitrogen atoms, respectively. The conformational change at the double bond between the nitrogen atoms results in a significant modification of the fitted coarse-grained molecular shape depicted by the transparent ellipsoids. Insets: locations of bond site positions at the GB ellipsoid surface and easy bond directions for the main-chain bonding (red) and for the optional cross-linking via the equatorial site (blue), as used in simulation. For the *trans* isomer, the head and tail bonding sites are assumed to impose an easy direction along the molecular long axis, while the optional site on the equator is taken to impose an easy direction along the second-longest molecular axis. In the *cis* isomer, the linking directions of the head/tail sites are tilted to make an angle of ≈46° with the long molecular axis.

The photophysics process of switching between the *trans* and *cis* states (see Figure 1) of an individual azobenzene molecule has been modeled in many studies in vacuum^4,5^ or, at the atomistic level, for azobenzene in isotropic solvents,^31,32^ but simulations performed at this level of detail can be considered only for systems with a small number of molecules. Atomistic and multiscale modeling has also been performed for the low molar mass azobenzene mesogen 4,4′-dioctyloxyazobenzene (8AB8) to investigate its phase changes.^33^ 7AB (*p*,*p*′-diheptylazobenzene) in 8CB (4-octyl-4′-cyanobiphenyl) has also been studied, in particular to examine the preferred location of *trans*- and *cis*-7AB in the smectic A phase of 8CB and finding that while the *trans* form is hosted inside the smectic layers, the *cis* tends to reside between the layers.^34^ Recently, atomistic simulations of azobenzenes in molecular- and polymer-network glasses have been carried out to study their behavior during and after photoactivation in a rigid matrix environment.^35,36^

As far as liquid crystal polymers are concerned, apart from theoretical efforts^37^ aimed at understanding azobenzene-based polymers, a few particle-based computer simulation studies have also been performed at the coarse-grained level,^38,39^ particularly with the purpose of studying the photoselective azobenzene excitation followed by effective reorientation of the director under linearly polarized UV light excitation.^14^

It is worth noting that, although very interesting in themselves, the details of the *trans–cis* conversion are not of central interest in terms of properties of the material exposed to isotropically distributed unpolarized UV light, if only for a time-scale argument. Indeed, the photoinduced *trans*-to-*cis* conversion is a very fast one that can even be performed in a few femtoseconds with a sufficiently powerful light source, while the rate of the spontaneous thermal *cis*-to-*trans* back conversion depends greatly on the specific compound^40^ and, unless pumped back by irradiation in the visible, where the typical *cis* absorption peak occurs, is typically slow to very slow (even well over a day^41^). We can then imagine that a system, initially with all azobenzenes in the *trans* state, could be suitably irradiated to yield a system with a certain controlled fraction of *cis* and that this system will be sufficiently long-lived to establish different molecular organizations according to the achieved fraction. Accordingly, our aim here is to investigate how the presence of this mixture of conformers will affect LCE phase transitions, sample deformation, and the differences of local structure around the two types of conformers. As far as we know, these aspects have not been dealt with before.

In the next sections, we describe our coarse-grained model for azobenzene LCEs and the range of Monte Carlo (MC) simulations performed on LCEs with various percentages of *trans* and *cis* conformers, monitoring ordering and structure as a function of temperature. Stress–strain curves at selected temperatures and *trans* and *cis* conformer ratio will also be provided. Conclusions will be collected in the last section.

## Model and Simulations

We choose a generic particle model where only the essentials of an azobenzene-based LCE are retained. This type of approach is probably unavoidable, considering that the actuation in LCE is a collective phenomenon involving a large number of particles. Moreover, reliable microscopic modeling of any cross-linked LCE system should involve samples whose size well exceeds the characteristic distance between the cross-links (that, in turn, significantly exceeds the size of a single molecule) to exclude the effects related to local irregularities in the LCE polymer network. Currently, sufficiently large sample sizes can only be achieved at the coarse-grained simulation level. Ideally, one would want to connect the benefits of the atomistic and coarse-grained treatment in a suitable multiscale approach. For this reason, in this study, we start from an atomistic-level optimization of the molecular structure for both azobenzene conformers, *trans* and *cis*, and use them to estimate the corresponding interaction parameters of the soft-core Gay–Berne (GB) attractive–repulsive potential acting between biaxial ellipsoidal particles representing the molecules^42,43^ that is commonly used in large-scale simulations of liquid-crystalline systems.^7^ With the thus obtained GB molecular building blocks, we assemble suitable LCE networks, following the methodologies developed in refs (44–46) and perform large-scale MC simulations. From the results, we determine the shift of the nematic–isotropic (NI) transition temperature upon increasing the *cis* particles content, together with the magnitude of the internal stress generated upon irradiation of the sample with UV light. Moreover, we examine the positional and orientational intermolecular correlations in the vicinity of both *trans* and *cis* particles.

We have previously modeled and simulated LCEs using soft GB units^47^ linked together by finitely extendable nonlinear elastic (FENE)^48,49^ spring-like bonds.^44−46^ The soft-core modification of the original GB (Appendix A)^42,50^ reduces its harsh repulsion, making entangling of the chains more manageable, and turns out to be essential for a proper equilibration of cross-linked GB polymer networks. In addition, a soft biaxial version of the GB potential (SBGB) has also been developed.^43^ It is thus natural to extend this approach to photosensitive LCEs by replacing some or all of monomer units with SBGBs modeling either *trans* or *cis* azobenzene moieties (see Figure 1a).

It is worth noting that an atomistic model, apart from being hardly feasible in terms of resources, as already mentioned, could even be not ideal, since it has to pay the appealing feature of being chemically realistic with the specificity of being applicable only to a certain chemical formulation. Since the actuation phenomenon we aim to better understand takes place for a variety of molecular structures, which have in common only the presence of the photoresponsive azobenzene moiety, it is desirable to develop a minimal model where only the *trans*–*cis* conversion is accounted for and other chemical details abandoned.

In practice, we study, using MC simulations, the effect of replacing a certain fraction of *trans* azobenzenes with *cis* conformers in the LCE. We shall go from full *trans* to full *cis*, considering fractions of *cisw*_c_ = 0, 0.2, 0.4, 0.6, 0.8, and 1.0 fixed during the simulation at various temperatures. In order to maximize the order-disruption effect and, consequently, the actuation, here all GB ellipsoids, both those of the polymer strands and the cross-linkers, are taken to be switchable azobenzenes as, for example, in ref (21). The coarse-graining step from the atomistic to coarse-grained description of a model azobenzene molecule is sketched in Figure 1b,c. As we can see, our minimal molecular model of an azobenzene photoresponsive unit consists of two aromatic rings connected via a N=N double bond whose loosening under the effect of UV light gives rise to the *trans–cis* isomerism^5,51^ that is responsible for the difference in the effective molecular shape. At the coarse-grained level, both isomers are represented by biaxial ellipsoids (since no molecule is perfectly uniaxial), whose interaction parameters need to be determined from an atomistic representation. Since we now have various types of units, interactions between dissimilar biaxial GB particles have to be considered.^52^

The main input parameters of the soft-core GB potential describing the two isomers are particle dimensions expressed through ellipsoidal axes (σ_*x*_, σ_*y*_, and σ_*z*_) and the energy parameters (ϵ_*x*_, ϵ_*y*_, and ϵ_*z*_) related to the attractive potential well depths for corresponding homogeneous particle alignments.^50^ Their values are reported in Table 1 and have been obtained from atomistic representations at united atom (UA), rather than at all-atom level. Note that not only the molecular aspect ratio and the relative characteristic potential well depths can be derived from the fitting but also their absolute values that eventually translate into macroscopically observable quantities. More details can be found in Appendix A and Appendix B.

**Table 1:** Soft-Core GB Potential Parameters (Ellipsoidal Axes in Units of σ_0_ = 0.35 nm and Energy Parameters in Units of ϵ_0_ = 1.3 × 10^–21^ J) for the Two Azo Isomers, as Obtained From the UA Fitting (Appendix B)

conformer	σ_*x*_	σ_*y*_	σ_*z*_	ϵ_*x*_	ϵ_*y*_	ϵ_*z*_
*trans*	1.00	1.21	4.00	1.00	0.25	0.08
*cis*	0.86	1.36	3.14	1.21	0.81	0.26

We consider a main-chain system where the elongated mesogenic moieties belong to the polymer backbone. In order to build an elastomer network, the fitted SBGB particles are bonded with other ellipsoids to build the main chains, as well as connected to other chains via cross-linking particles. In our minimal model, we neglect, apart from the change of shape, any realistic chemical details and just assume that each *trans* particle is decorated with a “head” and “tail” bonding site, that will be displaced at an angle in the *cis* molecule, following the conformational change. We also arbitrarily assume (ignoring at this idealistic model level any chemical difficulty) that the cross-linking particles have an additional bonding site on the equator. The positions of the bonding sites and the easy (preferred) directions of the resulting bonds, as assumed in the GB simulation, are indicated in the insets of Figure 1b,c.

The bonds themselves are modeled using the FENE potential^48^ applied both to bond stretching and bending,^44^ see Appendix A. The FENE potential, which is often used in modeling polymer chains, has the important feature of being stiffer for large deformations than its Hookean counterpart, imposing a maximum stretching and bending range.

All samples considered in this study contain *N* = 64,000 GB particles. Like in our previous work,^44−46^ we consider LCE swollen with a liquid-crystalline solvent (in practice a monomeric excess), which are characterized by a greater mobility of polymeric chains and have been experimentally studied.^53−55^ Thus, only one-half of the mesogens is used to build the elastomeric polymer network itself, while the rest represents the nonbonded swelling monomers introduced to facilitate the relaxation of polymer chains in simulation, while conveying nematicity at the same time.^44^ Periodic boundary conditions^56,57^ are applied in each sample preparation and in simulations to mimic a larger bulk sample. The polymer network is assembled following the steps similar to those presented in ref (46) to build polydomain LCE samples. Here, however, again to maximize actuation, we consider monodomain samples where the average molecular orientation, the nematic director, is the same throughout the sample and is imprinted parallel to, say, the laboratory *z*-axis. This is accomplished by growing, at low density, straight individual chains of particles, polydisperse and on average ∼12 monomers long, each starting from a random position within a cubic simulation box, directed at a random angle smaller than arccos(0.6) ≈ 53°, measured from the *z*-axis. Then, the active heads and tails of each chain are connected laterally to a nearest equatorial site on another, neighboring chain. A schematic representation of the typical resulting elastomer architecture is shown in Figure 1a. Finally, the system is soaked with swelling monomers and isotropically compressed almost to close-packing, increasing the density more than 20 times, to yield a cubic sample that we consider as a “reference” one. In principle, one should consider several of these reference samples to obtain a collection of LCE networks grown under the same conditions, but this is practically unfeasible in view of the computer time required. We have thus considered a single reference, also because, based on our previous experience,^44−46^ one replica for each *cis* particle concentration is sufficiently representative for a sample size as large as our one (*N* = 64,000) where the simulation box side significantly exceeds the average distance between the network cross-links.

Note that in our simulations, the reduced number densities , where *V* is the sample volume and σ_0_ is the shortest axis of the *trans* ellipsoid, depend on the *cis* particle fraction *w*_c_. This is because the fitted coarse-grained molecular volume (proportional to σ_*x*_σ_*y*_σ_*z*_) is somewhat smaller for the *cis* isomer than for the *trans* one (see Table 1), and it is necessary to avoid shifts of the NI transition due to a mere dilution effect. Consequently, the final sample densities increase with increasing *w*_c_. In a simplified approach, we determine these densities, summarized in Table 2, by keeping the molecular packing fraction unchanged for all *w*_c_. Note that the ρ* values employed are taken to be relaxed equilibrium values that are assumed constant during a simulation run. At variance with this, experiments^22^ have shown a reduction in sample density upon sample irradiation, which, however, is a transient phenomenon beyond the scope of the present study.

**Table 2:** Reduced Sample Densities ρ* as a Function of *cis* Particle Fraction *w*_c_

*w*_c_	0.0	0.2	0.4	0.6	0.8	1.0
ρ*	0.176	0.185	0.195	0.206	0.218	0.232

To find equilibrium configurations at a given *w*_c_ and temperature *T*, we performed MC simulations following the standard Metropolis algorithm.^7,58^ In each MC cycle (a cycle being a set of *N* attempted MC moves), particles are selected one by one in random order and undergo an individual trial move that is accepted if the associated interaction energy change Δ*U* is negative, or with a probability of exp(−Δ*U*/*k*_B_*T*) otherwise, where *k*_B_ denotes the Boltzmann constant. For a full definition of the interaction energy *U*, see Appendix A.

In each MC cycle, different trial move types are attempted separately: translational moves, rotational moves,^59^ and bonded pair rotations.^44^ In addition, every 5 MC cycles, a collective resize move is performed, where the cuboidal simulation box itself is randomly and affinely deformed at a constant volume, and subjected to the same Metropolis acceptance criterion as above (more details can be found in refs (44−46)). The trial move amplitudes are adjusted on the fly to maintain a trial move acceptance ratio of around 50% while ensuring that the system is evolving in terms of particle positions and orientations. In the calculations of interaction energies, the computational effort is reduced by setting up particle neighbor lists^60^ with interaction and list cutoffs at 6σ_0_ and 7σ_0_, respectively. Moreover, neighbor list updates are facilitated by means of cell-linked lists,^56^ and further parallelization schemes are employed to provide additional simulation speedup.

As a result, several 10^7^ MC cycles have typically been performed for sample equilibration and ∼10^6^ cycles for production. Typical averages of interest include (i) the reduced sample length λ_*z*_ along the *z*-axis, the nematic director of the monodomain, to monitor actuation, (ii) the standard nematic order parameter *P*_2_ to follow the underlying changes in orientational order, and (iii) the specific heat of the sample to facilitate the detection of the associated phase transitions. (i) Here, λ_*z*_ is conveniently defined as the average simulation box length along the *z*-axis, divided by the side of a reference sample containing *trans* particles only. (ii) The nematic order parameter calculation is performed calculating, for a certain MC cycle, the nematic ordering matrix
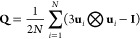
1where **u**_*i*_ stands for the long molecular axis of the *i*th molecule and **I** is a 3 × 3 identity matrix, and diagonalizing **Q** to identify its largest eigenvalue, which is then averaged over MC cycles to yield the order parameter indicated here as *P*_2_.^7^ (iii) Further, the reduced specific heat (per particle and in units of *k*_B_) is calculated as c* = d⟨U*⟩/dT*, where the reduced internal energy and temperature are introduced as U* = *U*/*N*ϵ_0_ and T* = *k*_B_*T*/ϵ_0_, respectively, and ⟨···⟩ stands for averaging over MC cycles. (Here, ϵ_0_ is the energy parameter related to the attractive GB potential well depth for a pair of perfectly aligned biaxial *trans* particles approaching along their shortest axes.^50,52^)

Finally, to identify layered smectic phases, the structure factor *S*(**q**) can be calculated as^61^
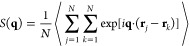
2where **q** denotes the scattering vector and **r**_*j*_ stands for the center-of-mass position of the *j*th anisotropic bead. Then, to assess the degree of smectic ordering, we define and calculate a smectic-like positional order parameter as , where **q**_1_ is parallel to the nematic director and corresponds to the first-order peak of *S*(**q**) resulting from smectic layering, similarly as in ref (62).

## Results and Discussion

In an aligned nematic containing *trans* azobenzene moieties either as part of the mesogens or as solutes, the presence of *cis* conformers represents a source of disruption in the alignment of their environment. This destabilizes the nematic phase and decreases the NI transition temperature, as observed experimentally.^8−14^ Therefore, we first explore the phase diagram of the LCE samples we have generated, as a function of reduced temperature T* and *cis* particle content, *w*_c_, even for very large values of the latter.

**Figure 2:**
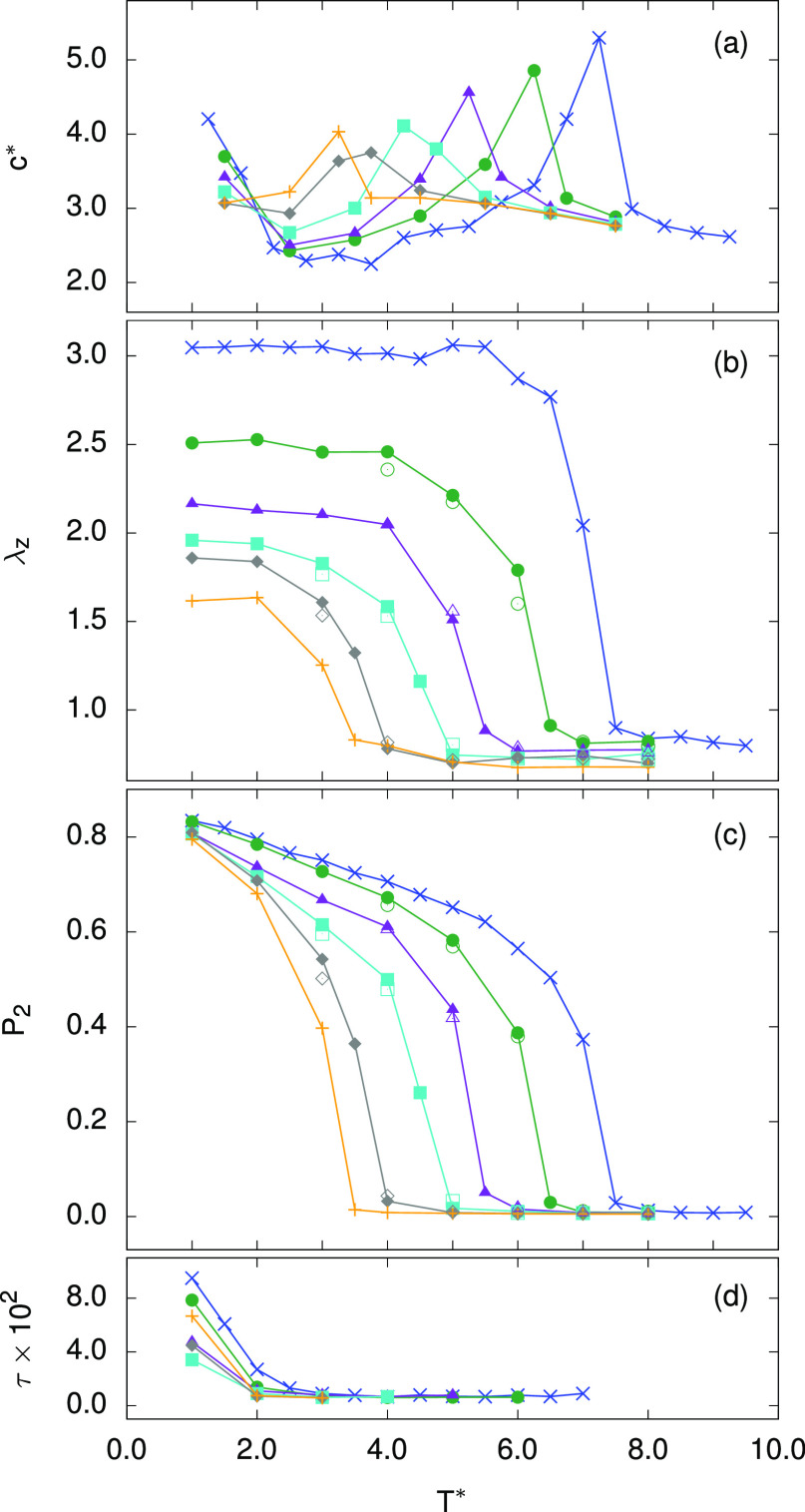
(a) Reduced constant volume specific heat per particle (c*), (b) sample length along the *z*-axis (λ_*z*_), as well as the (c) nematic and (d) smectic order parameters (*P*_2_ and τ, respectively), plotted as a function of reduced temperature for samples with different *cis* particle contents *w*_c_. The corresponding *cis* fractions are *w*_c_ = 0.0 (×), 0.2 (circles), 0.4 (triangles), 0.6 (squares), 0.8 (diamonds), and 1.0 (+). Additional data points, obtained for the same *w*_c_ but choosing a different set of *cis* particles, are also plotted for comparison (open symbols).

Simulation runs at all temperatures have been launched from the corresponding “reference” samples, except for a few cases at low temperatures (T* ≤ 3.0) where the runs were started from a pre-equilibrated configuration at a nematic temperature to facilitate MC equilibration. In an all-*trans* sample (*w*_c_ = 0), plots of the sample dimension λ_*z*_ and of the nematic order parameter *P*_2_ against temperature (Figure 2) reveal an isotropic-to-nematic phase transition at . (Taking ϵ_0_ ≈ 1.3 × 10^–21^ J, as obtained from the UA fitting, this yields a NI transition at ∼430 °C.) Below this temperature, the sample acquires an elongation along the *z*-axis, as imprinted upon sample preparation, that coincides with the nematic director, and a nonzero degree of nematic order (Figure 2b,c). The transition itself is also accompanied by a peak in the temperature dependence of the reduced specific heat, c* (Figure 2a). For this all-*trans* sample, there is also an additional peak in c* below T* ≈ 1.5 corresponding to the phase transition from the aligned nematic into the aligned and layered smectic phase—characterized by a nonzero smectic order parameter τ, Figure 2d—suffering from extremely slow MC relaxation.

As the *cis* particle content is increased, parallel nematic alignment is destabilized and the NI transition shifts toward lower temperatures as . (Taking again ϵ_0_ ∼ 1.3 × 10^–21^ J, the NI temperature is estimated to drop by ∼4.7 K for every additional % of sample particles excited into the *cis* state. Note, however, that a comparison with experiments is not straightforward here since both the NI transition temperature and its shift depend not only on the precise chemical nature of the mesogen but also on other features of the elastomer network and are thus quite system-dependent.) In a simulation, it is also possible to investigate the all-*cis* limit (*w*_c_ = 1.0), which may be rather far from reach with photoirradiation experiments, and from a nonzero *P*_2_ value (see Figure 2c), one can deduce that an aligned phase with orientational ordering is established even in this case. (Our simulations suggest that this happens for T* < 3.5 also in a nonbonded liquid-crystalline system consisting of monomers alone. In an LCE sample, such order is further enhanced by the mechanical aligning field^18^ imprinted along the *z*-axis upon sample preparation.)

We note that while working with a certain fraction *w*_c_ of *cis*-azobenzenes, the specific selection of *cis* particles is chosen (randomly) before the simulation and then left fixed. Ideally, one could consider many sample replicas with different *cis*–*trans* particle sets and perform an average over simulations of these isomeric replicas. In practice, each simulation for our sample size takes too long to make this feasible. However, to check the robustness of our results, typically obtained from a single isomeric replica, we have repeated the temperature-scan experiment on a second alternative sample with a different *cis*-particle distribution (and the same *w*_c_) and the results for the two replicas considered (especially the  values) turn out to be in very good agreement (see Figure 2 and compare full and empty symbols).

The presence of shorter *cis* particles locally disrupts the nematic orientational order established by the more anisotropic *trans* particles. To gain more insight into how this happens, it is instructive to study the surroundings of each isomer type separately by calculating their radial distribution functions *g*(*r*_*ij*_) and orientational order correlation functions *G*_2_(*r*_*ij*_), for example, from a single molecular snapshot. *g*(*r*_*ij*_) is calculated by identifying and counting all particle pairs where the first particle belongs to the selected azo-species and then creating a corresponding histogram with respect to the interparticle distance *r*_*ij*_. The histograms are normalized with respect to an uncorrelated uniform fluid and then averaged over all possible selections of the first particle of the given species. As such, *g*(*r*_*ij*_) is then proportional to the local density of all particles surrounding a particle of a given azo-species. A similar analysis can be performed by averaging *P*_2_(**u**_*i*_·**u**_*j*_) over eligible pairs of particles *i* and *j* (instead of just counting the pairs), where **u**_*i*_ and **u**_*j*_ denote the particle long axes and *P*_2_(*x*) = (3*x*^2^ – 1)/2 is the second Legendre polynomial, giving the second-rank orientational pair correlation^7^
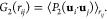
3

**Figure 3:**
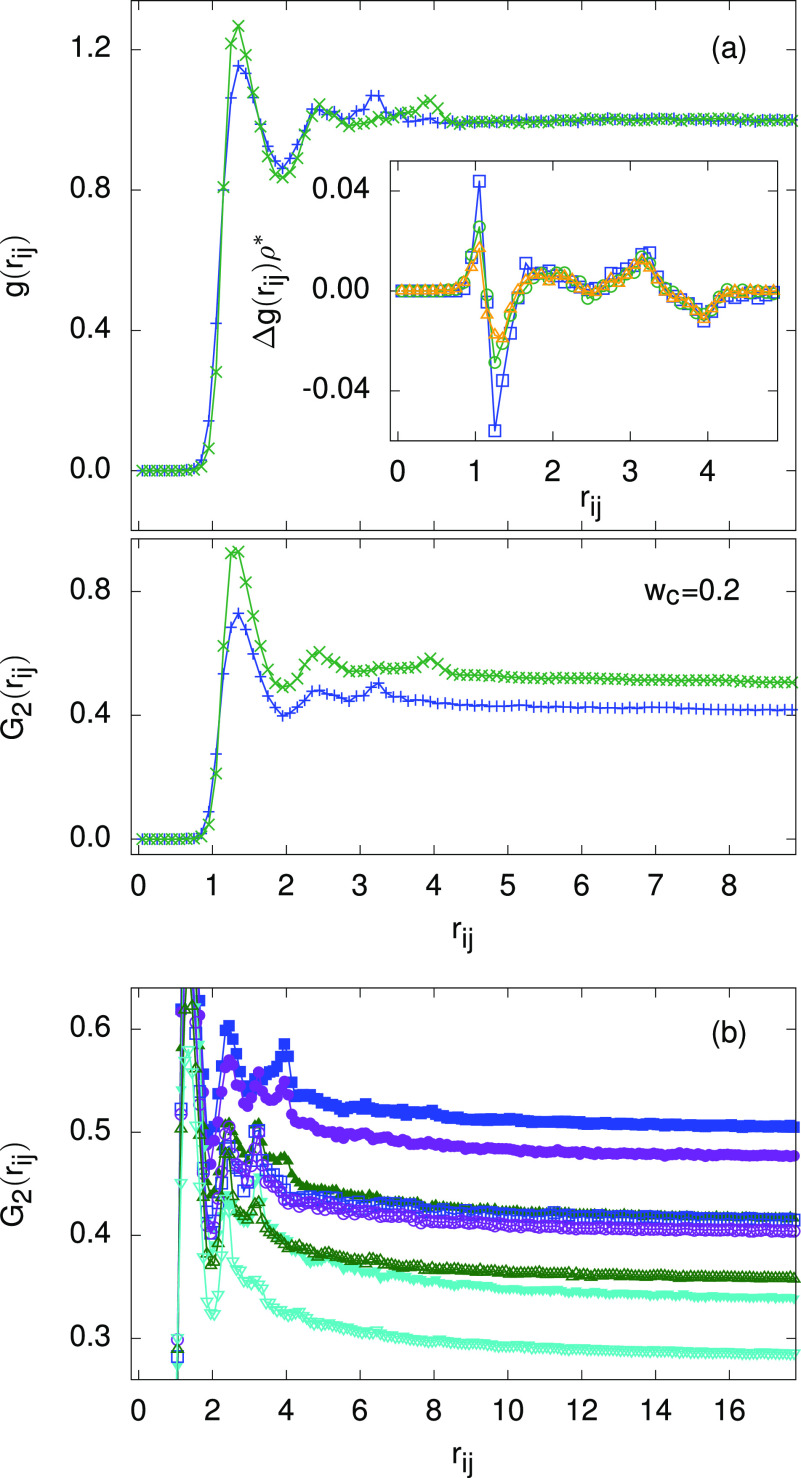
Pair correlation properties. (a) Top: radial distribution functions *g*_c_(*r*_*ij*_) (+) and *g*_t_(*r*_*ij*_) (×) at T* = 3.0 and *w*_c_ = 0.2, plotted separately for the surroundings of *cis* and *trans* particles, respectively. Bottom: same as above but showing the corresponding orientational correlation functions *G*_2_(*r*_*ij*_). Inset: the difference of the local densities surrounding the two particle types (see text) at T* = 2.0 (squares), 3.0 (circles), and 4.0 (triangles), for *w*_c_ = 0.2. (b) *G*_2_(*r*_*ij*_) correlation functions at T* = 3.0 for the surroundings of *cis* and *trans* particles (open and closed symbols, respectively) for *w*_c_ = 0.2 (squares), *w*_c_ = 0.4 (circles), *w*_c_ = 0.6 (triangles up), and *w*_c_ = 0.8 (triangles down). The interparticle distances *r*_*ij*_ are plotted in units of σ_0_.

The correlation properties in a sample with 20% *cis*-content are summarized in Figure 3a. The radial distribution functions *g*_c_(*r*_*ij*_) and *g*_t_(*r*_*ij*_) for the surroundings of *cis* and *trans* isomers (top plate), respectively, show no major qualitative differences at a nematic temperature of T* = 3.0, but the details are compatible with the somewhat different particle shapes (see Table 1). The difference of average local particle densities in the vicinity of *cis* and *trans* particles, given by Δ*g*(*r*_*ij*_)ρ*, where Δ*g*(*r*_*ij*_) = *g*_c_(*r*_*ij*_) – *g*_t_(*r*_*ij*_) and ρ* is the average sample density, is shown as inset in Figure 3a at different T* in the aligned nematic phase. At smallest distances, *g*_c_(*r*_*ij*_) > *g*_t_(*r*_*ij*_) due to the smaller σ_*x*_ for the *cis* isomer, which is followed by a pronounced *g*_c_(*r*_*ij*_) < *g*_t_(*r*_*ij*_) region. As a result, within a small sphere ∼4σ_0_ in diameter surrounding a *cis* particle, there are slightly less molecules than in an equal-sized sphere surrounding a *trans* particle. This appears to be consistent with observations and hypotheses by Liu.^63^ Recall that here *w*_c_ = 0.2, so that the vast majority of isomers are in *trans* state. A *cis* particle thus represents a slight ordering disruption and is somewhat less efficiently embedded into its predominantly *trans*-environment, than any *trans* particle itself. From the inset of Figure 3a, it also follows that these effects are more pronounced at lower temperatures.

Further, the orientational correlation functions *G*_2_(*r*_*ij*_) are shown for *w*_c_ = 0.2 in the bottom plate of Figure 3a separately for both isomers. At small *r*_*ij*_, they show a similar structure as their *g*(*r*_*ij*_) counterparts, while for large *r*_*ij*_ where the short-range correlations are already lost, they are expected to relax to a value equal to the product of the nematic order parameters of the central (*cis* or *trans*) and a far-away (any type) azobenzene particle. (This follows from the addition theorem for second-order spherical harmonics in the absence of biaxial molecular ordering.) From the lower *G*_2_(*r*_*ij*_ → ∞) value for a *cis* particle at the center in comparison with the corresponding value for a central *trans* particle, one can conclude that the degree of orientational order of *cis* particles themselves (in a *trans*-rich *w*_c_ = 0.2 sample) is lower than that of the *trans* particles. Similar behavior is also systematically observed at larger *w*_c_ values (see Figure 3b): the longer *trans* particles are always more orientationally ordered than the shorter *cis* ones, which results in a higher *G*_2_(*r*_*ij*_ → ∞) value in case of a central *trans* particle, compared with that of a central *cis* particle. At the same time, the *G*_2_(*r*_*ij*_ → ∞) values, at given temperature, for both particle types decrease with increasing *w*_c_ due to a general weakening of the nematic ordering (Figure 2c).

**Figure 4:**
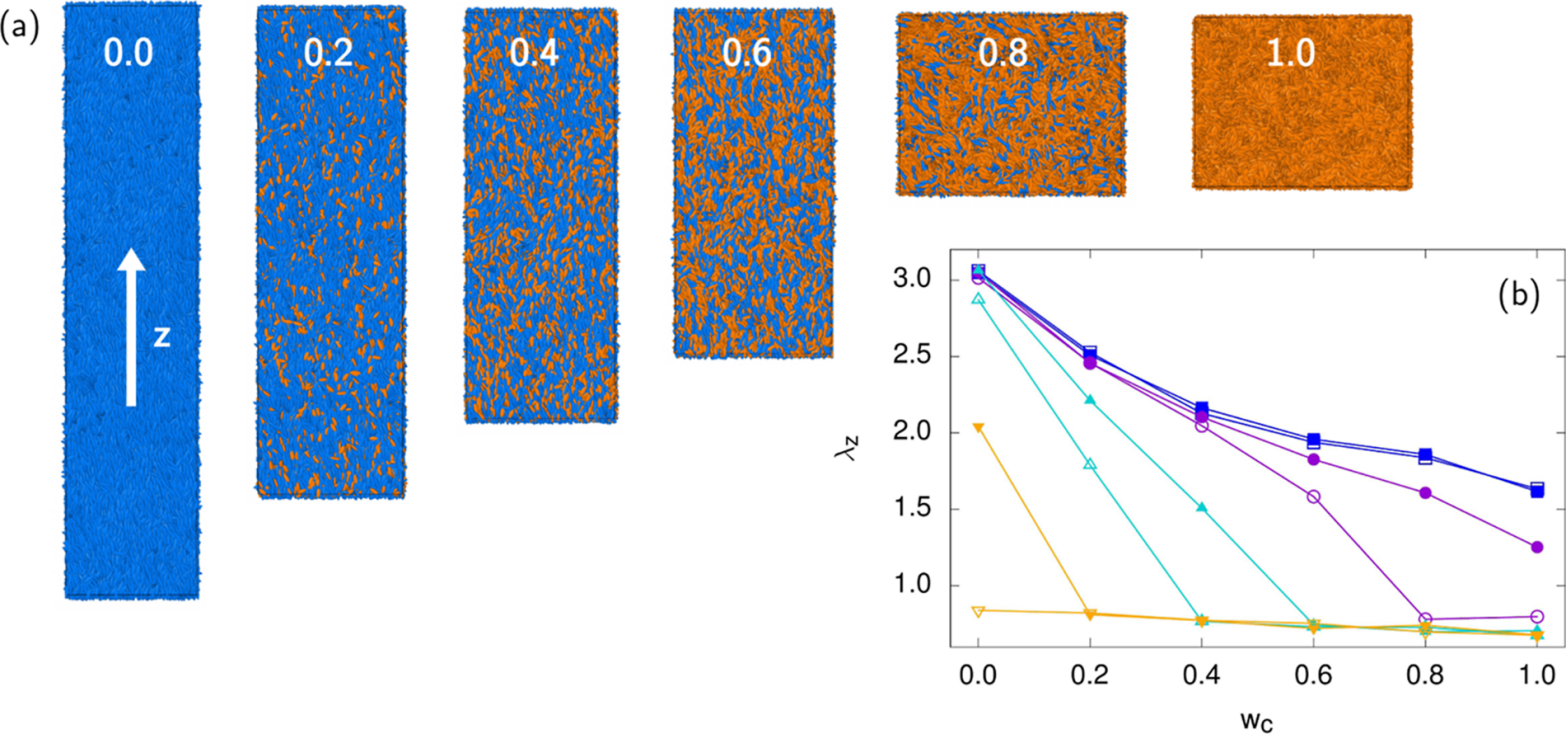
(a) Molecular snapshots depicting a gradual irradiation of an LCE sample at T* = 4.0, with corresponding *cis* particle fractions *w*_c_ displayed. *Trans* particles are colored blue, and *cis* particles orange. Upon *cis* fraction increasing, nematic orientational order is lost and the sample contracts along the director, *z* axis. (b) Reduced sample length along the *z*-axis, λ_*z*_, plotted against *w*_c_ for different temperatures: T* = 1.0 (closed squares), 2.0 (open squares), 3.0 (closed circles), 4.0 (open circles), 5.0 (closed triangles up), 6.0 (open triangles up), 7.0 (closed triangles down), and 8.0 (open triangles down).

Studying photoresponsive LCE, it is also interesting to look at sample behavior when increasing the *w*_c_ fraction at fixed temperature. Figure 4a shows a series of molecular snapshots in such a situation at T* = 4.0 where the actuative effect upon changing *w*_c_ is largest (as can be deduced from Figure 2). At this temperature, an all-*trans* (*w*_c_ = 0) sample is nematically ordered and the sample strongly elongated along the *z*-axis. The increase of *cis*-content, *w*_c_, results in a gradual destruction of orientational order, as well as in the loss of sample elongation. This behavior could be exploited in actuation to extract useful mechanical work from the system. The simulation box side λ_*z*_ vs *w*_c_ is shown as inset, Figure 4b, for different temperatures. All dependences decrease monotonically, whereby lower temperatures generally result in a higher value of λ_*z*_.

**Figure 5:**
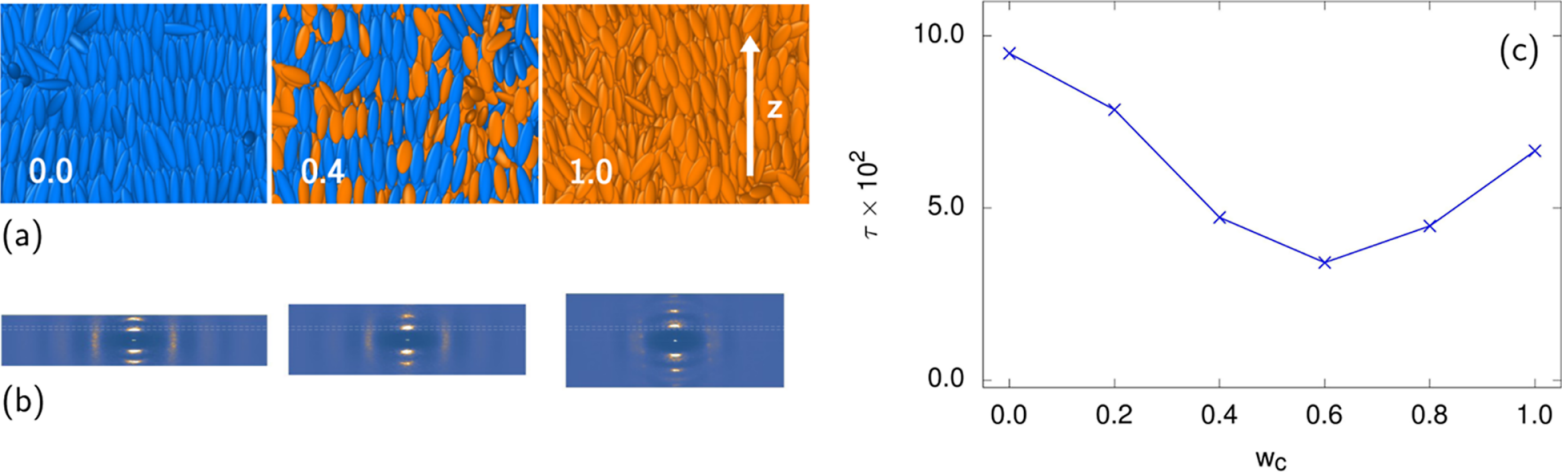
(a) Close-up snapshots for *w*_c_ = 0 (all-*trans*), *w*_c_ = 0.4, and *w*_c_ = 1.0 (all-*cis*) in the smectic phase (T* = 1.0), together with the corresponding structure factor patterns (b). Same molecular color coding as in Figure 4a is used. The two dashed horizontal hairlines in plots (b) indicate the position of the first maximum associated with smectic layering in the all-*trans* and all-*cis* samples. (c) Smectic order parameter τ as a function of *w*_c_. Smectic layering is partially disrupted as the *cis*-particle content increases, but the orientational ordering and sample elongation are preserved even for *w*_c_ = 1.0. In an all-*cis* sample, the smectic ordering is to some extent restored.

At low enough temperatures, the simulated LCE samples are in the smectic phase. Given that in an LCE system, the ellipsoidal molecules are bonded in a rather complex cross-linked network, the smectic layers are well-defined but present plenty of defects. The molecular snapshot close-ups depicting actuation as *w*_c_ increases in a simulated smectic LCE at T* = 1.0 are shown in Figure 5a. The increasing presence of the short *cis* particles additionally perturbs the smectic layers initially consisting of longer *trans* particles, but the orientational order, together with sample elongation, persists even in an all-*cis* sample at *w*_c_ = 1.0. In addition to molecular organization snapshots (Figure 5a), the presence of positional smectic ordering is further evidenced by the structure factor patterns depicted in Figure 5b where a series of peaks along the *z*-axis (parallel to the smectic layer normal and the nematic director) is clearly visible. (The less pronounced arcs in the direction perpendicular to *z* are a signature of interlayer molecular ordering.) Figure 5c further shows the evolution of the smectic order parameter τ with increasing *cis*-content: at first, τ decreases due to the somewhat disrupted layering. However, approaching the all-*cis* sample, τ increases again, which can be attributed to the absence of the perturbing longer *trans* particles in a *cis*-rich environment. Note also that with increasing *w*_c_, the structure factor peaks move to a slightly higher wavevector value |**q**_1_|, compatibly with the shorter long axis of *cis* particles.

Above we have seen that upon light-induced actuation, an initially elongated sample contracts along the nematic director, *z*-axis. Such behavior applies to a free (unclamped) sample. Alternatively, one could also consider an irradiation experiment with a clamped sample whose length λ_*z*_ is fixed. A *trans–cis* photoisomerization under such conditions is inevitably accompanied by a generation of internal stress resulting from an increase of molecular disorder that is, in turn, balanced by the external stress of the clamps. To estimate the magnitude of stress thus generated at a given temperature, we consider a contracted sample with *w*_c_ ≠ 0 and apply external stress to stretch the sample along the *z*-axis to its initial length λ_*z*_ measured at *w*_c_ = 0. Each stretching experiment is performed through an isostress MC run^57^ where, apart from temperature, engineering stress Σ_*zz*_ along the *z*-axis (here calculated with respect to the surface area corresponding to a face of the all-*trans* reference sample) is fixed. Having fixed an additional extensive thermodynamic variable, the Metropolis acceptance criterion has to be modified: the internal energy change Δ*U* is replaced by the change of enthalpy Δ*H*,^57,64^ where the interaction enthalpy *H* is given by *U* – Σ_*zz*_*V*λ_*z*_; *V* here denotes the corresponding sample volume. The reduced external engineering stress is conveniently defined as .

**Figure 6:**
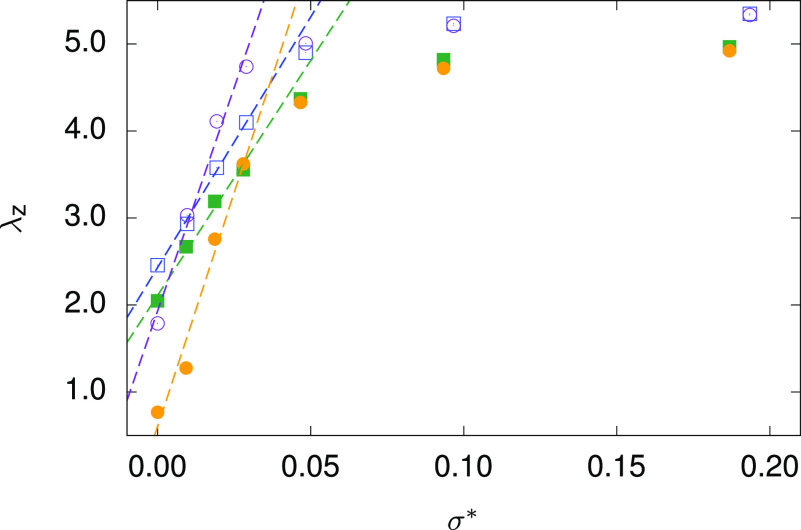
Stress–strain curves used for the estimation of the internal stress generated upon sample irradiation with light. The open symbols correspond to *w*_c_ = 0.2, the closed ones to *w*_c_ = 0.4; the squares correspond to T* = 4.0, the circles to T* = 6.0. Dotted lines represent the linear fits in the low-stress region (σ* < 0.03).

It is quite impossible to guess the correct value of stress σ* necessary to stretch an irradiated sample far enough to exactly match its initial length. For this reason, we simulate full stress–strain curves λ_*z*_(σ*) and perform the necessary extrapolation between the data points thus obtained to find σ*. Each of the necessary simulation runs was started from a sample previously equilibrated at the required conditions (T*, *w*_c_) and zero stress. In Figure 6, stress–strain curves for two values of *w*_c_ (0.2 and 0.4) and two temperatures (4.0 and 6.0) are plotted. In the low-stress region, σ* < 0.03, the dependence is close to linear and, as a byproduct, the corresponding isothermal Young elastic moduli can be estimated from the λ_*z*_(σ*) slopes determined by appropriate fitting. Taking ϵ_0_ ∼ 1.3 × 10^–21^ J and σ_0_ ∼ 0.35 nm from the UA fitting, the resulting values at T* = 4.0 are ∼3.3 and ∼2.6 MPa for *w*_c_ = 0.2 and *w*_c_ = 0.4, respectively. At a higher temperature of T* = 6.0, ∼1.0 and ∼0.2 MPa are estimated for *w*_c_ = 0.2 and *w*_c_ = 0.4, respectively. The modulus decreases with increasing *cis* particle content and decreases with temperature; its values are relatively high but compatible with our previous results for simulated LCE in the nematic phase.

Turning now to the values of internal stress σ* in a clamped sample, at T* = 4.0, we obtain 0.010 and 0.017 for *w*_c_ = 0.2 and *w*_c_ = 0.4, respectively. At T* = 6.0, the corresponding values are 0.009 and 0.021. (Assuming the same values for ϵ_0_ and σ_0_ as above, the given σ* values are to be multiplied by ∼30 MPa to obtain the engineering stress Σ_*zz*_.) At a given temperature, internal stress in a clamped sample thus increases with irradiation (leading to higher *cis*-content *w*_c_) since a larger sample contraction has to be compensated for by the clamps. For the same reason, at a given *w*_c_, one would expect internal stress to increase also with increasing temperature, but this is not always the case since at higher temperature our samples also become softer, which reflects in a reduction of the Young’s elastic modulus. Finally, the typical magnitude of the generated internal stress is estimated to be of the order of several hundred kPa.

## Conclusions

We have performed a computational study of the effects that different percentages of *trans* and *cis* azobenzene monomeric units can have on the properties of a model LCE. More specifically, a large number of coarse-grained MC computer simulations (over 80) have been employed to study shape changes in monodomain main-chain LCEs formed by azobenzene moieties with various fractions of *trans* and *cis* conformers. The photoresponsive moieties were represented by soft-core GB biaxial ellipsoids whose interaction parameters were obtained from a fit of the UA-level interaction energy for selected pair configurations. Our results reveal a decrease of the NI transition temperature upon increasing *cis* population (e.g., by increased sample irradiation). In samples with prevailing *trans*-particle presence, the immediate neighborhood of *cis* particles is slightly less dense than that of the *trans* ones that are more compatible with their average environment. Simulations of light-induced actuation at fixed temperature reveal a significant contraction of the sample along the monodomain director, which could be exploited to perform useful mechanical work. This effect is most pronounced when actuating at temperatures not too far below the NI transition. Finally, from the simulated stress–strain curves, we conclude that in a clamped sample, internal mechanical stress of the order of several hundred kPa can be generated upon irradiation with light and that this stress increases with the content of *cis*-isomers, but not necessarily with temperature.

## Appendix A

### Coarse-Grained Pair Potentials

As mentioned in the text, the coarse-grained pair potentials considered in our study are the soft-core biaxial GB (SBGB) for the nonbonded interactions and the finitely extensible nonlinear potential (FENE) for the bonded ones.

The biaxial soft-core GB potential^43^ acting between dissimilar ellipsoidal particles *i* and *j* is defined as^47^

4

where  is the linear repulsive soft-core potential and  is the biaxial GB potential.^50,52^ The sigmoidal logistic function  switches smoothly between the soft-core repulsion at small interparticle distances *r*_*ij*_ and the attractive potential well of the GB potential at larger *r*_*ij*_. Above, *m* and *n* are parameters (we use *m* = −70ϵ_0_/σ_0_ and *n* = −100/σ_0_^44−46^), and σ_*ij*_ is the anisotropic distance function for the two particles given by . Here , where **r**_*ij*_ is the interparticle vector and *r*_*ij*_ = |**r**_*ij*_|. The distance function σ_*ij*_ also depends on the relative particle orientation, which is encoded into the **A**_*ij*_ overlap matrix defined by , where **M**_*i*_ is the rotation matrix that brings any vector from the laboratory reference frame to that of molecule *i*.^65^ Moreover, **S**_*i*_ are range matrices describing the molecular shape, with the corresponding σ_*x*_, σ_*y*_, and σ_*z*_ ellipsoidal axes on the diagonal and zeros elsewhere.

The biaxial GB potential is now given by

5

where σ_*ij*_ and *r*_*ij*_ were defined above and σ_c_ denotes the minimum interparticle contact distance. (In the case of dissimilar particles, an arithmetic average of the shortest axes of the particles is taken.^52^) Further, , where ϵ_0_ sets the energy scale and μ and ν are empirical constants. (We consider μ = 1 and ν = 3 for compatibility with previous work.) Moreover, here , with . Finally, the attractive potential well anisotropy is incorporated into , where , and **E**_*i*_ are diagonal matrices with elements , , and , where ϵ_*x*_, ϵ_*y*_, and ϵ_*z*_ are related to the well depths for the homogeneous interactions in characteristic orthogonal molecular configurations (see refs (50) and (52)).

We employ the FENE potential^48^ for interparticle bonds to describe both their stretching and bending. Consider a bond connecting two neighboring ellipsoids *i* and *j*, with a length of *s*_*ij*_. Then, the deviation from its equilibrium value *s*_e_ equals δ*s*_*ij*_ = |*s*_*ij*_ – *s*_e_|, and let us assume that δ*s*_*ij*_ < δ*s*_m_. (There is a limitation on stretching.) Similarly, the bond angle θ_*ij*_ is defined as the angle between the preferred bond directions imposed by each of the particles.^66^ The deviation from its equilibrium value θ_e_ is δθ_*ij*_ = |θ_*ij*_ – θ_e_|, with a requirement that δθ_*ij*_ < δθ_m_. (There is a limitation on bending, too.) Now, the FENE bond energy is given by^66,67^

6

the first contribution referring to bond stretching and the second one to bond bending. The values of the potential parameters introduced above are *s*_e_ = 0.15σ_0_, δ*s*_m_ = 0.25σ_0_, and  for stretching, and θ_e_ = 0°, δθ_m_ = 150°, and *k*_θ_ = 3.8 × 10^–4^ϵ_0_/deg^2^ ≈ 1.72 × 10^–21^ J/rad^2^ for bending.^44−46,66,67^

The total interaction energy *U* for a system of *N* particles is obtained by summing up all nonbonded and bonded intermolecular pairwise contributions, , where *w*_*ij*_ = 1 if the monomers *i* and *j* are bonded, and *w*_*ij*_ = 0 otherwise.

## Appendix B

### United-Atom Fitting of Azobenzene Conformers

We performed a simple energy minimization of atomistic structures of the two isomers of azobenzene with “Argus Lab”^68^ choosing the semiempirical Quantum Chemistry (QC) method AM1 (Austin model 1) and the Restricted Hartree–Fock self-consistent field method (for closed shells).^69^ We recall that we are only interested in getting the basic shape change upon *trans–cis* photoisomerization of the bare bone azobenzene unit in Figure 1, thus using more sophisticated QC methods is unnecessary, if not inappropriate. Using the force field published in ref (31), we have obtained molecular ellipsoids overdimensioned with respect to the atomistic structures. Hence, we have applied a UA approach, maintaining the same nomenclature of the force field but including into the carbon atom of the benzene ring the hydrogen attached to it.^7^ Atomic masses and charges have been modified accordingly to take into account the collapsed hydrogens. The dimensions of the biaxial molecular ellipsoid are computed using the “shape tensor” **T**_s_, a modified version of the inertia matrix in which the masses are substituted with the van der Waals radii^70^ to consider steric hindrance of the atomic centers, as reported below

7

Here, *r*_*i*_ is the van der Waals radius of the *i*th atom and the total sum of radii is denoted by *r*_T_ = ∑_*i*_*r*_*i*_. The eigenvalues λ_*xx*_, λ_*yy*_, and λ_*zz*_ of **T**_s_ are related to the dimensions of the ellipsoid and can be used to define its axes as follows

8

9

10

When the molecule is flat (as in the trans isomer), the length of the shortest axis approaches zero. Therefore, the standard distance of π–π stacking, 0.35 nm, has been used instead.

The energy parameters ϵ_*x*_, ϵ_*y*_, and ϵ_*z*_ related to the potential well depths are computed using the atomistic structure and the UA force field described above. Two identical molecules have been placed at different distances, and their interaction energy within the chosen force field has been computed. The interaction energy has been obtained as a function of intermolecular distance for the three most significant pair configurations: side-by-side, face-to-face, and end-to-end. These profiles have then been fitted with the soft-core GB potential, using the ellipsoid dimensions obtained from the shape tensor.

The parameters summing up fitting results for the *cis* and *trans* UA azobenzene conformers represented as prolate GB ellipsoids are reported in the text (Table 1).

## References

[ref1] Zhao Y.; Ikeda T. Smart Light-Responsive Materials. Azobenzene Containing Polymers and Liquid Crystals; Wiley: Hoboken, NJ, 2009.

[ref2] Ravve A. Light-Associated Reactions of Synthetic Polymers; Springer: New York, 2006, p 246.

[ref3] Bertrand O.; Gohy J.-F. Photo-responsive polymers: synthesis and applications. Polym. Chem. 2017, 8, 52–73. 10.1039/c6py01082b.

[ref4] Cembran A.; Bernardi F.; Garavelli M.; Gagliardi L.; Orlandi G. On the mechanism of the cis-trans isomerization in the lowest electronic states of azobenzene: S0, S1, and T1. J. Amer. Chem. Soc. 2004, 126, 3234–3243. 10.1021/ja038327y. 15012153

[ref5] Aleotti F.; Soprani L.; Nenov A.; Berardi R.; Arcioni A.; Zannoni C.; Garavelli M. Multidimensional potential energy surfaces resolved at the RASPT2 Level for accurate photoinduced isomerization dynamics of azobenzene. J. Chem. Theory Comput. 2019, 15, 6813–6823. 10.1021/acs.jctc.9b00561. 31647648

[ref6] de Gennes P. G.; Prost J. The Physics of Liquid Crystals; Oxford University Press: Oxford, 1993.

[ref7] Zannoni C. Liquid Crystals and Their Computer Simulations; Cambridge University Press: Cambridge, 2022.

[ref8] Kurihara S.; Ikeda T.; Tazuke S. Photochemically induced isothermal phase transition in liquid crystals. Effect of interaction of photoresponsive molecules with matrix mesogens. Mol. Cryst. Liq. Cryst. 1990, 178, 117–132. 10.1080/00268949008042713.

[ref9] Ikeda T.; Horiuchi S.; Karanjit D. B.; Kurihara S.; Tazuke S. Photochemically induced isothermal phase-transition in polymer liquid crystals with mesogenic phenyl benzoate side-chains. 1. Calorimetric studies and order parameters. Macromolecules 1990, 23, 36–42. 10.1021/ma00203a008.

[ref10] Ikeda T.; Horiuchi S.; Karanjit D. B.; Kurihara S.; Tazuke S. Photochemically induced isothermal phase-transition in polymer liquid crystals with mesogenic phenyl benzoate side-chains. 2. Photochemically induced isothermal phase-transition behaviors. Macromolecules 1990, 23, 42–48. 10.1021/ma00203a009.

[ref11] Ikeda T.; Kurihara S.; Karanjit D. B.; Tazuke S. Photochemically induced isothermal phase-transition in polymer liquid crystals with mesogenic cyanobiphenyl side-chains. Macromolecules 1990, 23, 3938–3943. 10.1021/ma00219a012.

[ref12] Tsutsumi O.; Shiono T.; Ikeda T.; Galli G. Photochemical phase transition behavior of nematic liquid crystals with azobenzene moieties as both mesogens and photosensitive chromophores. J. Phys. Chem. B 1997, 101, 1332–1337. 10.1021/jp961565d.

[ref13] Vecchi I.; Arcioni A.; Bacchiocchi C.; Tiberio G.; Zanirato P.; Zannoni C. Expected and unexpected behavior of the orientational order and dynamics induced by azobenzene solutes in a nematic. J. Phys. Chem. B 2007, 111, 3355–3362. 10.1021/jp0651788. 17388493

[ref14] Ichimura K. Photoalignment of liquid crystal systems. Chem. Rev. 2000, 100, 1847–1874. 10.1021/cr980079e. 11777423

[ref15] Fang G. J.; Maclennan J. E.; Yi Y.; Glaser M. A.; Farrow M.; Korblova E.; Walba D. M.; Furtak T. E.; Clark N. A. Athermal photofluidization of glasses. Nat. Commun. 2013, 4, 1521 10.1038/ncomms2483. 23443549

[ref16] Yadav B.; Domurath J.; Kim K.; Lee S.; Saphiannikova M. Orientation approach to directional photodeformations in glassy side-chain azopolymers. J. Phys. Chem. B 2019, 123, 3337–3347. 10.1021/acs.jpcb.9b00614. 30896167

[ref17] Warner M.; Terentjev E. M. Liquid Crystal Elastomers; Oxford University Press: Oxford, 2003.

[ref18] de Gennes P. G. Réflexions sur un type de polymères nématiques (Some reflections about a type of nematic liquid crystal polymers). C. R. Acad. Sc. Paris 1975, 281, 101–103.

[ref19] White T. J. Photomechanical effects in liquid crystalline polymer networks and elastomers. J. Polym. Sci. B 2018, 56, 695–705. 10.1002/polb.24576.

[ref20] Camacho-Lopez M.; Finkelmann H.; Palffy-Muhoray P.; Shelley M. Fast liquid-crystal elastomer swims into the dark. Nat. Mater. 2004, 3, 307–310. 10.1038/nmat1118. 15107840

[ref21] Yu Y. L.; Nakano M.; Ikeda T. Directed bending of a polymer film by light - Miniaturizing a simple photomechanical system could expand its range of applications. Nature 2003, 425, 145 10.1038/425145a. 12968169

[ref22] Liu D. Q.; Broer D. J. New insights into photoactivated volume generation boost surface morphing in liquid crystal coatings. Nat. Commun. 2015, 6, 8334 10.1038/ncomms9334. 26388022 PMC4595720

[ref23] White T. J. Photomechanical Materials, Composites, and systems: Wireless Transduction of Light Into Work, 1st ed.; White T. J., Ed.; Wiley: Hoboken, NJ, 2017; pp 153–178.

[ref24] Photomechanical Materials, Composites, and Systems: Wireless Transduction of Light into Work; White T. J., Ed.; Wiley: Hoboken, 2017.

[ref25] Zheng X. X.; Jia Y. N.; Chen A. H. Azobenzene-containing liquid crystalline composites for robust ultraviolet detectors based on conversion of illuminance-mechanical stress-electric signals. Nat. Commun. 2021, 12, 4875 10.1038/s41467-021-25178-2. 34385464 PMC8360969

[ref26] Ceamanos L.; Kahveci Z.; López-Valdeolivas M.; Liu D. L.; Broer D. J.; Sánchez-Somolinos C. Four-Dimensional Printed Liquid Crystalline Elastomer Actuators with Fast Photoinduced Mechanical Response toward Light-Driven Robotic Functions. ACS Appl. Mater. Interfaces 2020, 12, 44195–44204. 10.1021/acsami.0c13341. 32885661

[ref27] Shimamura A.; Priimägi A.; Mamiya J.-i.; Ikeda T.; Yu Y.; Barrett C. J.; Shishido A. Simultaneous Analysis of Optical and Mechanical Properties of Cross-Linked Azobenzene-Containing Liquid-Crystalline Polymer Films. ACS Appl. Mater. Interfaces 2011, 3, 4190–4196. 10.1021/am200621j. 22017368

[ref28] Zeng H.; Wasylczyk P.; Wiersma D. S.; Priimägi A. Microrobotics: Light Robots: Bridging the Gap between Microrobotics and Photomechanics in Soft Materials. Advanced Materials 2018, 30, 1870174 10.1002/adma.201870174. 29067734

[ref29] Kotikian A.; McMahan C.; Davidson E. C.; Muhammad J. M.; Weeks R. D.; Daraio C.; Lewis J. A. Untethered soft robotic matter with passive control of shape morphing and propulsion. Sci. Robot. 2019, 4, eaax7044 10.1126/scirobotics.aax7044. 33137783

[ref30] Herbert K. M.; Fowler H. E.; McCracken J. M.; Schlafmann K. R.; Koch J. A.; White T. J. Synthesis and alignment of liquid crystalline elastomers. Nat. Rev. Mater. 2021, 7, 23–38. 10.1038/s41578-021-00359-z.

[ref31] Tiberio G.; Muccioli L.; Berardi R.; Zannoni C. How does the trans–cis photoisomerization of azobenzene take place in organic solvents?. ChemPhysChem 2010, 11, 1018–1028. 10.1002/cphc.200900652. 20235111

[ref32] Cantatore V.; Granucci G.; Persico M. Simulation of the π → π* photodynamics of azobenzene: Decoherence and solvent effects. Comput. Theor. Chem. 2014, 1040–1041, 126–135. 10.1016/j.comptc.2014.02.011.

[ref33] Böckmann M.; Marx D.; Peter C.; Delle Site L.; Kremer K.; Doltsinis N. L. Multiscale modelling of mesoscopic phenomena triggered by quantum events: light-driven azo-materials and beyond. Phys. Chem. Chem. Phys. 2011, 13, 7604–7621. 10.1039/c0cp01661f. 21267491

[ref34] Lansac Y.; Glaser M. A.; Clark N. A.; Lavrentovich O. D. Photocontrolled nanophase segregation in a liquid-crystal solvent. Nature 1999, 398, 54–57. 10.1038/17995.

[ref35] Salerno K. M.; Lenhart J. L.; de Pablo J. J.; Sirk T. W. Vapor-deposited glasses highlight the role of density in photostability. J. Phys. Chem. B 2020, 124, 6112–6120. 10.1021/acs.jpcb.0c03579. 32609518

[ref36] Salerno K. M.; Lenhart J. L.; de Pablo J. J.; Sirk T. W. Photoisomerization and local stability in molecular and polymer-network glasses. Mol. Syst. Des. Eng. 2023, 8, 105–114. 10.1039/D2ME00092J.

[ref37] Toshchevikov V.; Saphiannikova M. Theory of light-induced deformation of azobenzene elastomers: Effects of the liquid crystalline interactions and biaxiality. J. Phys. Chem. B 2014, 118, 12297–12309. 10.1021/jp5063226. 25254355

[ref38] Ilnytskyi J. M.; Saphiannikova M. Reorientation dynamics of chromophores in photosensitive polymers by means of coarse-grained modeling. ChemPhysChem 2015, 16, 3180–3189. 10.1002/cphc.201500500. 26272323

[ref39] Ilnytskyi J. M.; Toshchevikov V.; Saphiannikova M. Modeling of the photo-induced stress in azobenzene polymers by combining theory and computer simulations. Soft Matter 2019, 15, 9894–9908. 10.1039/c9sm01853k. 31774109

[ref40] García-Amorós J.; Velasco D. Recent advances towards azobenzene-based light-driven real-time information-transmitting materials. Beilstein J. Org. Chem. 2012, 8, 1003–1017. 10.3762/bjoc.8.113. 23019428 PMC3458718

[ref41] Zhou L.; Chen L.; Ren G.; Zhu Z.; Zhao H.; Wang H.; Zhang W.; Han J. Monitoring cis-to-trans isomerization of azobenzene using terahertz time-domain spectroscopy. Phys. Chem. Chem. Phys. 2018, 20, 27205–27213. 10.1039/C8CP04570D. 30345440

[ref42] Gay J. G.; Berne B. J. Modification of the overlap potential to mimic a linear site-site potential. J. Chem. Phys. 1981, 74, 3316–3319. 10.1063/1.441483.

[ref43] Berardi R.; Lintuvuori J. S.; Wilson M. R.; Zannoni C. Phase diagram of the uniaxial and biaxial soft–core Gay–Berne model. J. Chem. Phys. 2011, 135, 134119 10.1063/1.3646310. 21992294

[ref44] Skačej G.; Zannoni C. Main-chain swollen liquid crystal elastomers: a molecular simulation study. Soft Matter 2011, 7, 9983–9991. 10.1039/c1sm05709j.

[ref45] Skačej G.; Zannoni C. Molecular simulations elucidate electric field actuation in swollen liquid crystal elastomer. Proc. Natl. Acad. Sci. U.S.A. 2012, 109, 10193–10198. 10.1073/pnas.1121235109. 22679288 PMC3387105

[ref46] Skačej G.; Zannoni C. Molecular simulations shed light on supersoft elasticity in polydomain liquid crystal elastomers. Macromolecules 2014, 47, 8824–8832. 10.1021/ma501836j.

[ref47] Berardi R.; Zannoni C.; Lintuvuori J. S.; Wilson M. R. A soft-core Gay-Berne model for the simulation of liquid crystals by Hamiltonian replica exchange. J. Chem. Phys. 2009, 131, 174107 10.1063/1.3254019. 19894998

[ref48] Bird R. B.; Curtiss C. F.; Armstrong R. C.; Hassager D. Dynamics of polymeric liquids; John Wiley & Sons: New York, 1987; Vol. 2, p 21.

[ref49] Khare R. S.; de Pablo J. J.; Yethiraj A. Rheology of confined polymer melts. Macromolecules 1996, 29, 7910–7918. 10.1021/ma960083x.

[ref50] Berardi R.; Fava C.; Zannoni C. A generalized Gay-Berne intermolecular potential for biaxial particles. Chem. Phys. Lett. 1995, 236, 462–468. 10.1016/0009-2614(95)00212-m.

[ref51] Persico M.; Granucci G. Photochemistry: A Modern Theoretical Perspective; Springer: Cham, 2018, pp 23–128.

[ref52] Berardi R.; Fava C.; Zannoni C. A Gay-Berne potential for dissimilar biaxial particles. Chem. Phys. Lett. 1998, 297, 8–14. 10.1016/s0009-2614(98)01090-2.

[ref53] Urayama K.; Honda S.; Takigawa T. Electrooptical effects with anisotropic deformation in nematic gels. Macromolecules 2005, 38, 3574–3576. 10.1021/ma0503054.

[ref54] Fukunaga A.; Urayama K.; Koelsch P.; Takigawa T. Electrically driven director-rotation of swollen nematic elastomers as revealed by polarized Fourier transform infrared spectroscopy. Phys. Rev. E 2009, 79, 051702 10.1103/PhysRevE.79.051702. 19518469

[ref55] Urayama K.; Takigawa T. Cross-Linked Liquid Crystalline Systems: from Rigid Polymer Networks to Elastomers; Broer D., Crawford G. P., Žumer S., Eds.; CRC Press, Taylor & Francis: Boca Raton, 2011; pp 473–486.

[ref56] Allen M. P.; Tildesley D. Computer Simulations of Liquids, 2nd ed.; Clarendon Press: Oxford, 2017; pp 35–195.

[ref57] Frenkel D.; Smit B. Understanding Molecular Simulation: From Algorithms to Applications; Academic Press: San Diego, 1996; pp 28–112.

[ref58] Metropolis N.; Rosenbluth A. W.; Rosenbluth M. N.; Teller A. H.; Teller E. Equation of state calculations by fast computing machines. J. Chem. Phys. 1953, 21, 1087–1092. 10.1063/1.1699114. 41342507

[ref59] Barker J. A.; Watts R. O. Structure of water; A Monte Carlo calculation. Chem. Phys. Lett. 1969, 3, 144–145. 10.1016/0009-2614(69)80119-3.

[ref60] Verlet L. Computer ”experiments” on classical fluids. I. Thermodynamical properties of Lennard-Jones molecules. Phys. Rev. 1967, 159, 98–103. 10.1103/physrev.159.98.

[ref61] Chaikin P. M.; Lubensky T. C. Principles of Condensed Matter Physics; Cambridge University Press: Cambridge, 1995, p 33.

[ref62] Polson J. M.; Frenkel D. First-order nematic-smectic phase transition for hard spherocylinders in the limit of infinite aspect ratio. Phys. Rev. E 1997, 56, R6260–R6263. 10.1103/PhysRevE.56.R6260.

[ref63] Liu D. Q. Volume generation towards dynamic surface morphing in liquid crystal polymer networks. Liq. Cryst. 2016, 43, 2136–2143. 10.1080/02678292.2016.1195898.

[ref64] Callen H. B. Thermodynamics and an Introduction to Thermostatistics; John Wiley & Sons: New York, 1985, p 147.

[ref65] Rose M. E. Elementary Theory of Angular Momentum; Wiley: New York, 1957, p 48.

[ref66] Berardi R.; Micheletti D.; Muccioli L.; Ricci M.; Zannoni C. A computer simulation study of the influence of a liquid crystal medium on polymerization. J. Chem. Phys. 2004, 121, 9123–9130. 10.1063/1.1790453. 15527380

[ref67] Micheletti D.; Muccioli L.; Berardi R.; Ricci M.; Zannoni C. Effect of nano-confinement on liquid crystal polymer chains. J. Chem. Phys. 2005, 123, 224705 10.1063/1.2125707. 16375493

[ref68] Thompson M. A. Molecular Docking Using ArgusLab, an Efficient Shape-Based Search Algorithm and the AScore Scoring Function; ACS Meeting: Philadelphia, 2004.

[ref69] Cramer C. J. Essentials of Computational Chemistry. Theories and Models; Wiley: New York, 2004, pp 126–145.

[ref70] Bondi A. Van der Waals volumes and radii. J. Phys. Chem. 1964, 68, 441–451. 10.1021/j100785a001.

[ref71] Stukowski A. Visualization and analysis of atomistic simulation data with OVITO–the Open Visualization Tool. Modell. Simul. Mater. Sci. Eng. 2010, 18, 015012 10.1088/0965-0393/18/1/015012.

